# Supplementary material for: Immunizing lithium metal anodes against dendrite growth using protein molecules to achieve high energy batteries
Source: Nat Commun. 2020 Oct 27;11:5429. doi: 10.1038/s41467-020-19246-2 (PMC7591880; doi:10.1038/s41467-020-19246-2)
Supplement: Supplementary file 1 — Supplementary Information [file 41467_2020_19246_MOESM1_ESM.pdf]

Supplementary Information

Immunizing lithium metal anodes against dendrite growth using protein molecules to achieve high energy batteries

Wang et al.

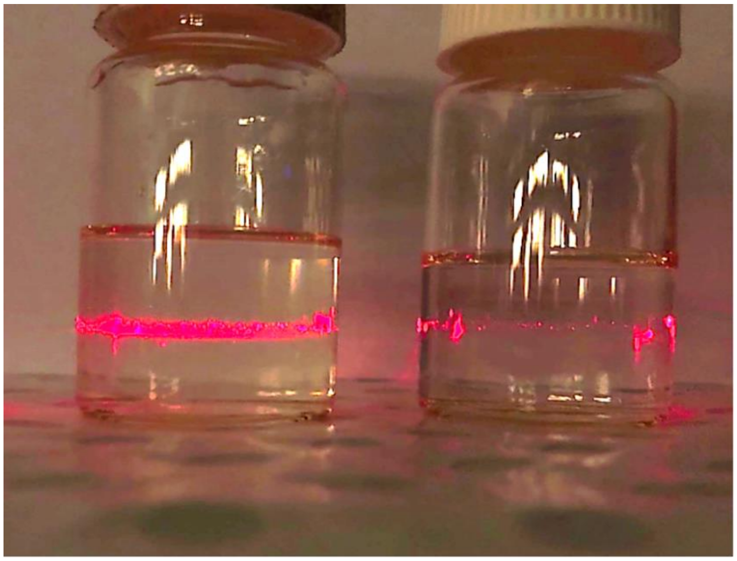

Supplementary Figure 1. Digital photo of Tyndall effect in ether-based electrolytes with and without fibroin.

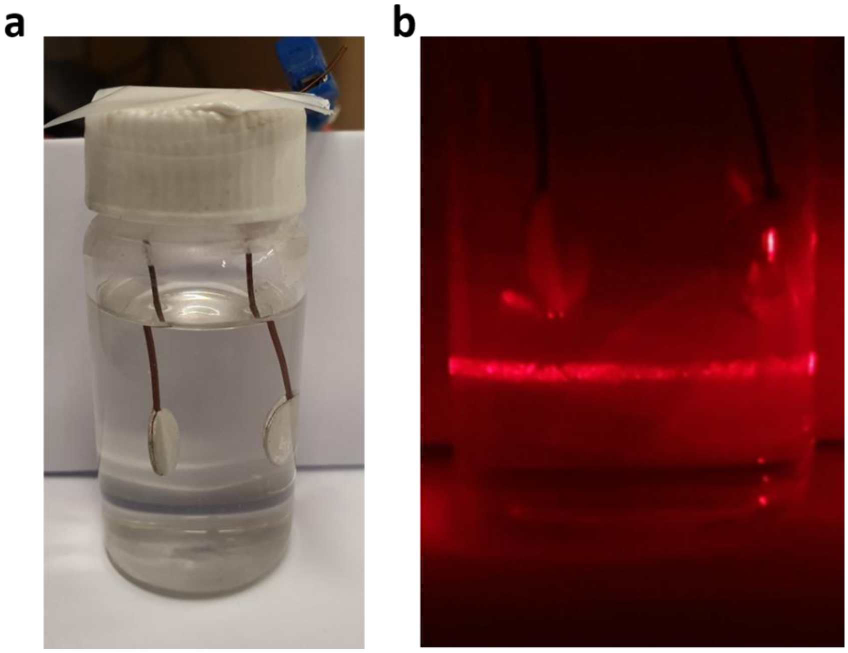

Supplementary Figure 2. Tyndall effect of fibroin dispersed electrolyte during Li | Li plating/stripping process. Tyndall effect can be observed in a transparent Li | Li symmetric cell during plating/stripping process at a current density of 1 mA cm^{-2}, which confirms that fibroin molecules are dispersed homogeneously in the electrolyte during cycling.

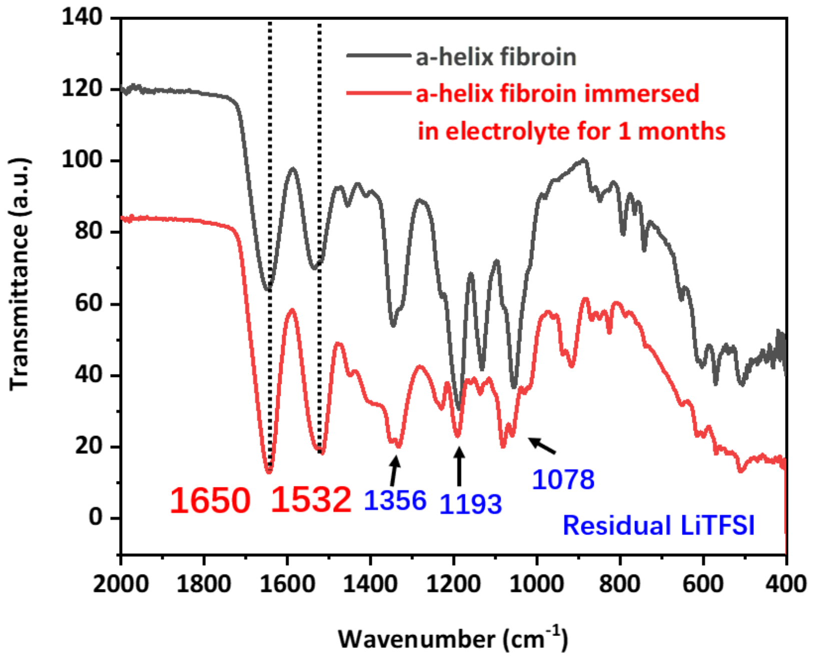

Supplementary Figure 3. Comparison of FT-IR curves of fibroin before and after immersed in the ether-based electrolyte for 1 month.

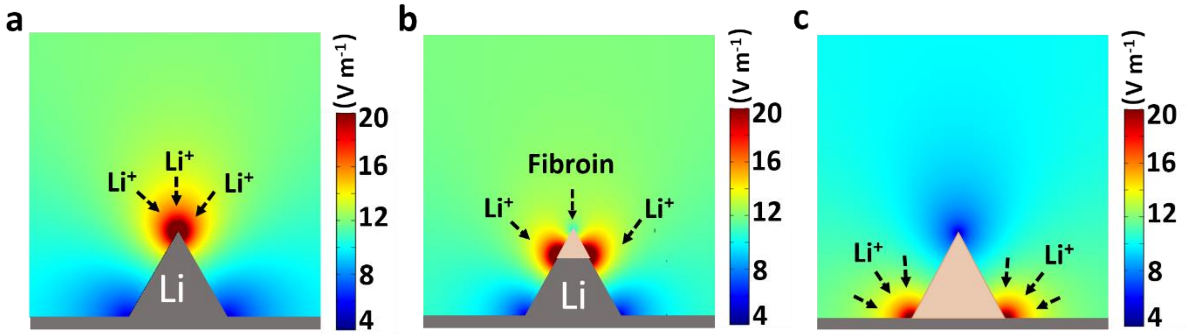

Supplementary Figure 4. COMSOL™ simulation of the change of electric field distribution around Li buds. a before and **b**, **c** after covered by fibroin molecules with the height ratio of (**b**) 30% and (**c**) 100%.

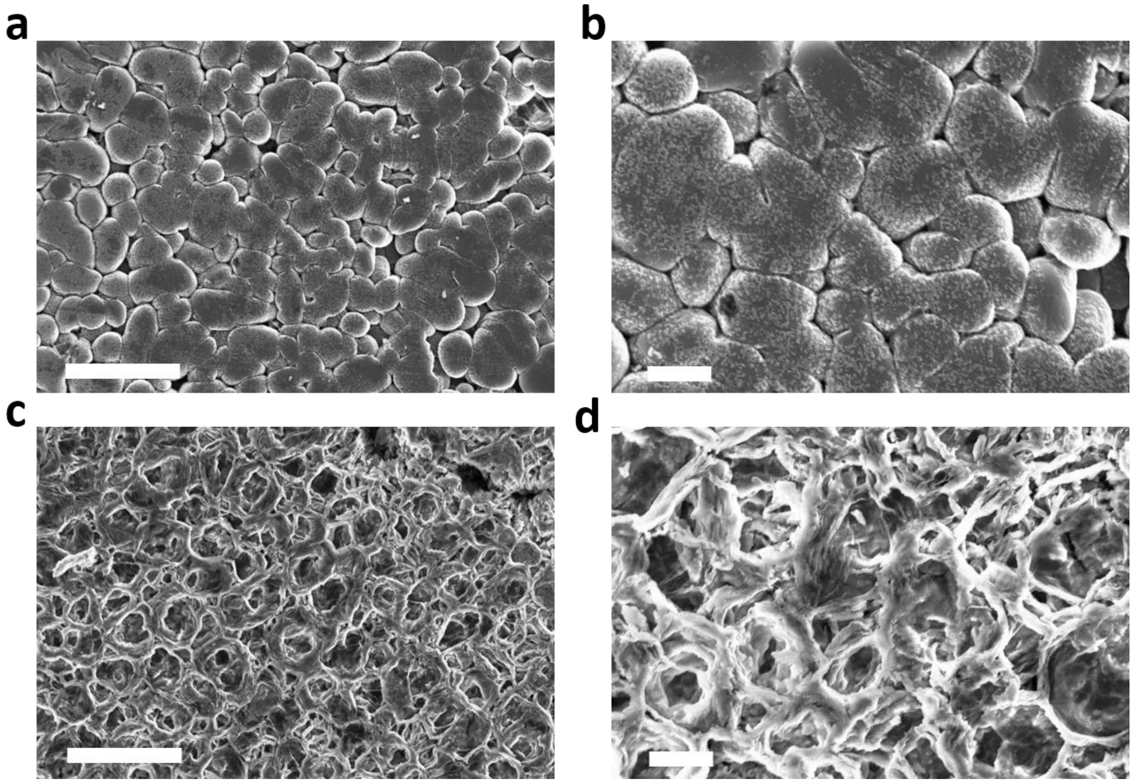

Supplementary Figure 5. SEM images of Li metal surface after 100 cycles. a, b In the electrolyte with fibroin. **c, d** In the electrolyte without fibroin. The current density is 1 mA cm^{-2} and the capacity limitation is 1 mAh cm^{-2}. Scale bar, (a, c) 10 μm and (b, d) 2 μm.

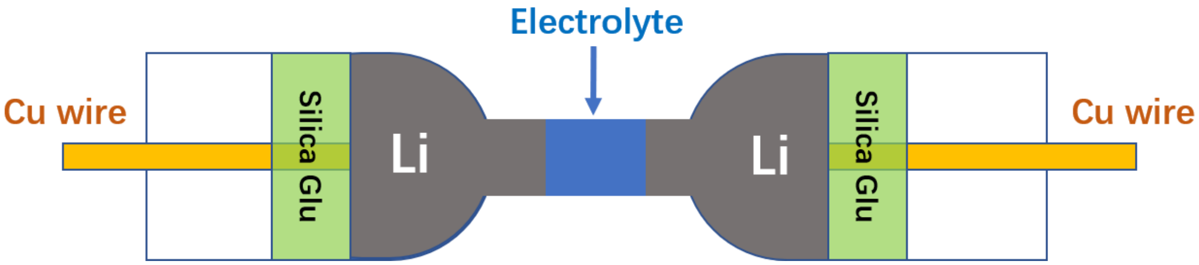

Supplementary Figure 6. Schematic illustration of the configuration of Li | Li symmetric cells assembled in a homemade glass capillary.

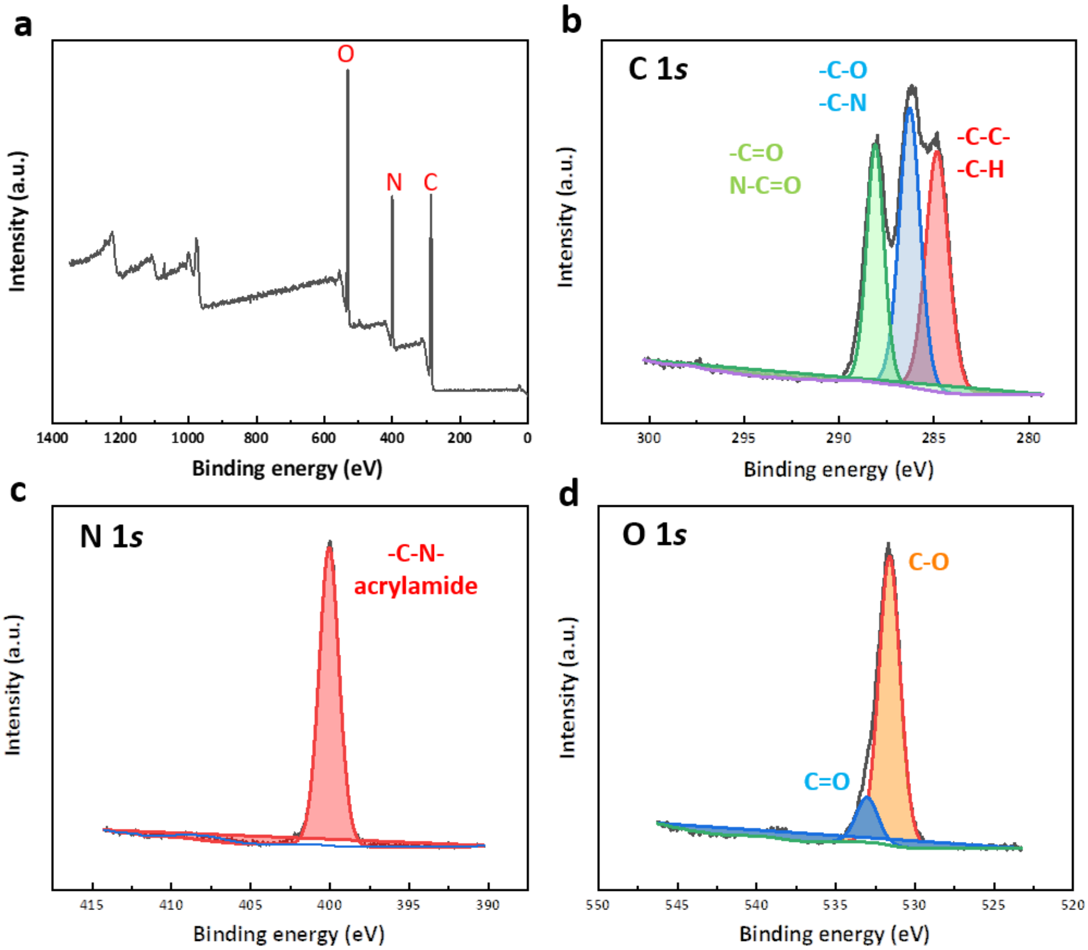

Supplementary Figure 7. XPS spectra of pristine fibroin. **a** Survey spectrum. **b** C1*s* spectra. **c** N1*s* spectra. **d** O1*s* spectra.

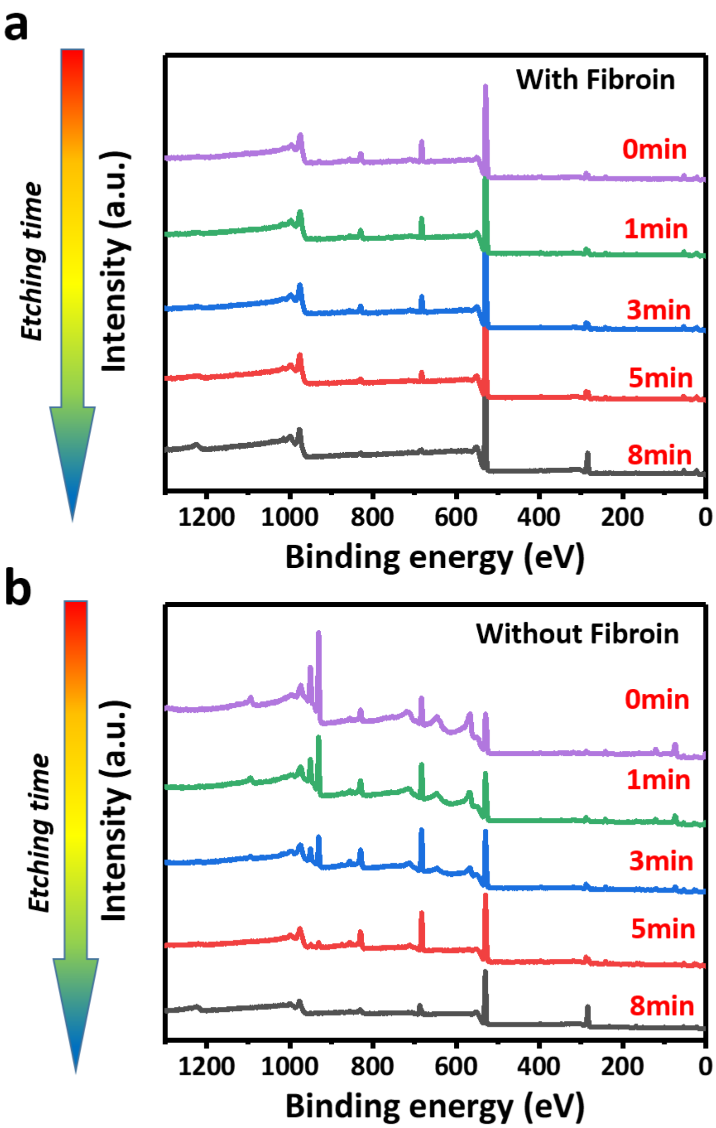

Supplementary Figure 8. XPS spectra of cycled Li metal anode. XPS survey spectra of the SEI after different degrees of etching by Ar-ion sputtering **a** with and **b** without fibroin additive in the ether-based electrolyte.

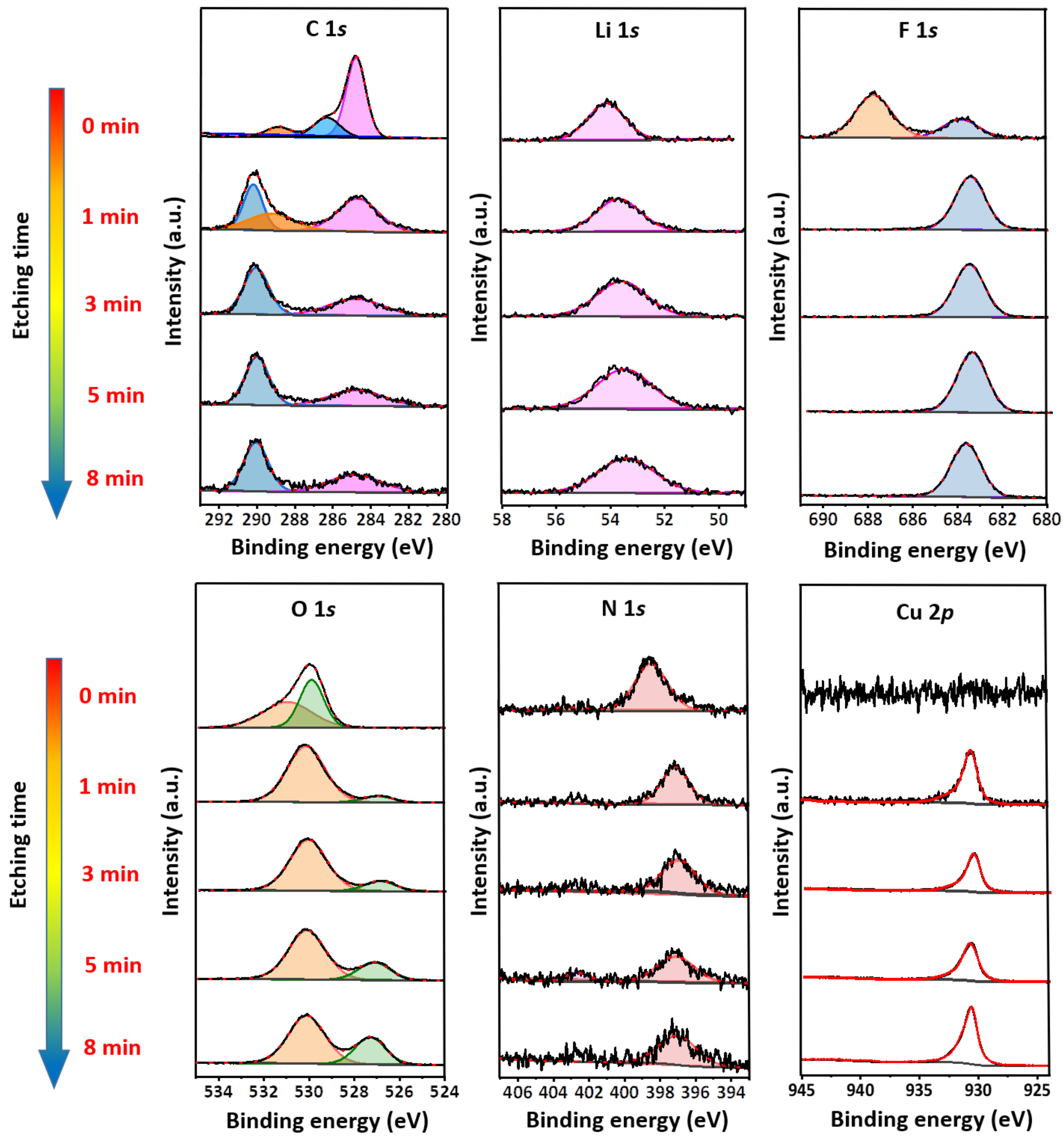

Supplementary Figure 9. In-depth XPS profiles of the SEI formed in ether-based electrolyte without fibroin. Li deposition was carried out at a current density of 1mA cm^{-2} and a capacity of 1 mAh cm^{-2} on Cu foil for 10 cycles.

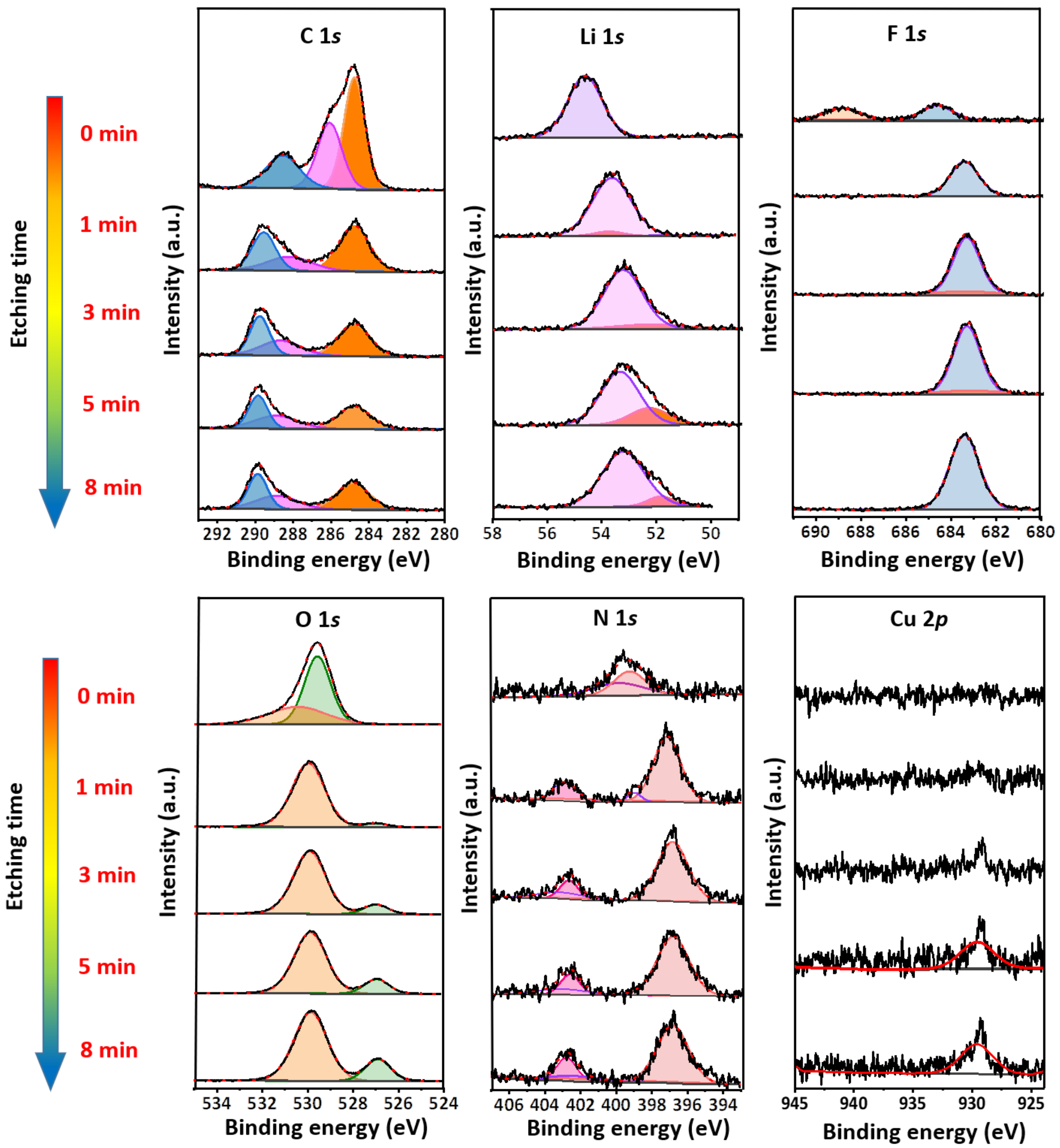

Supplementary Figure 10. In-depth XPS profiles of the SEI formed in ether-based electrolyte with fibroin. Li deposition was carried out at a current density of 1mA cm^{-2} and a capacity of 1 mAh cm^{-2} on Cu foil for 10 cycles.

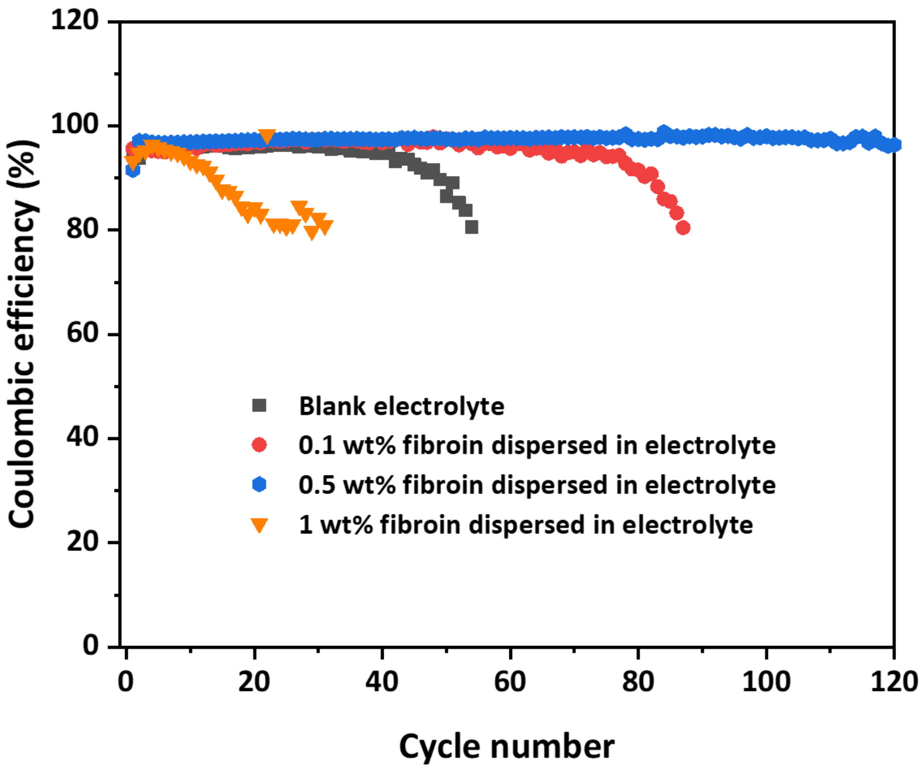

Supplementary Figure 11. Cycling stability of Li | Cu half cells with ether-based electrolyte containing different concentrations of fibroin.

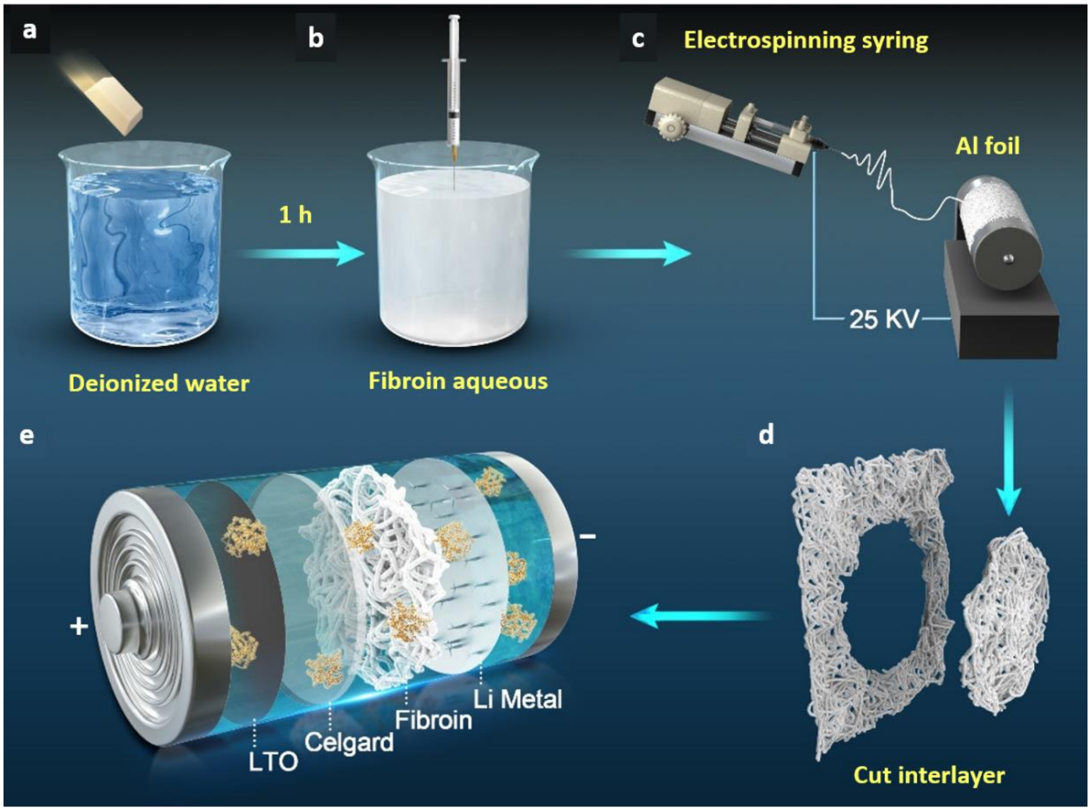

Supplementary Figure 12. Schematic illustration of the synthesis process of fibroin interlayer. **a-d** Schematic illustration of electrospinning system for preparing a non-woven fibroin interlayer. **e** The architecture of Li metal battery using $Li_4Ti_5O_{12}$ (LTO) as cathode, one piece of fibroin interlayer between the Celgard™ separator and Li metal anode.

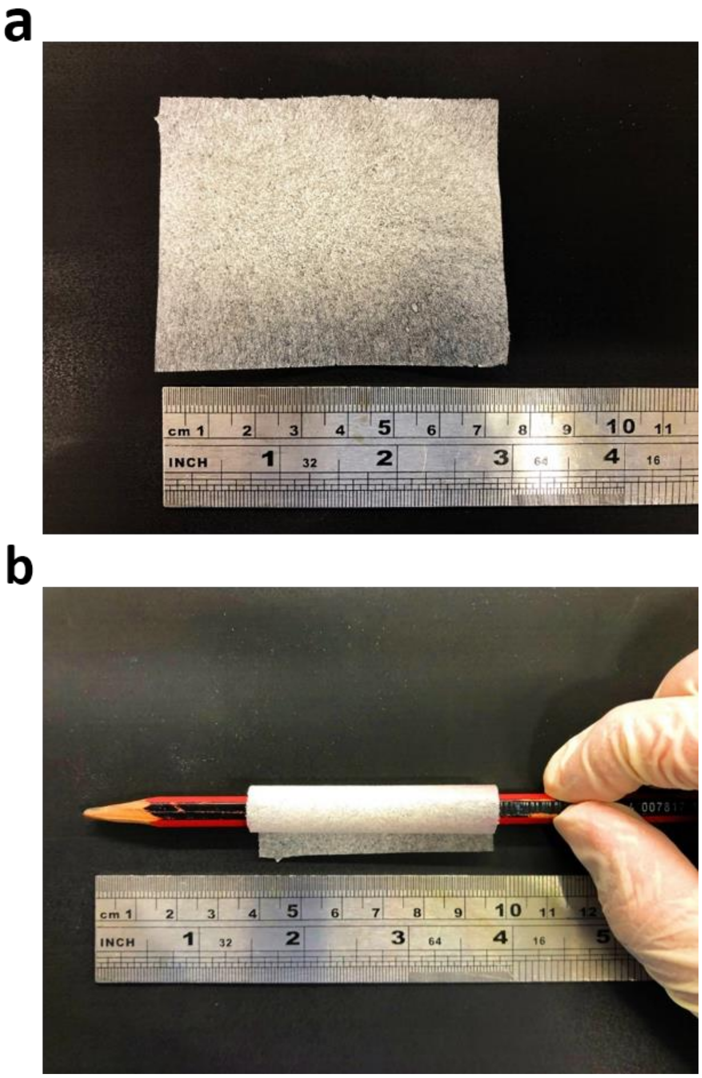

Supplementary Figure 13. Digital photos of fibroin interlayer. a A piece of flat fibroin interlayer. **b** A piece of flexible fibroin interlayer wrap around a pencil.

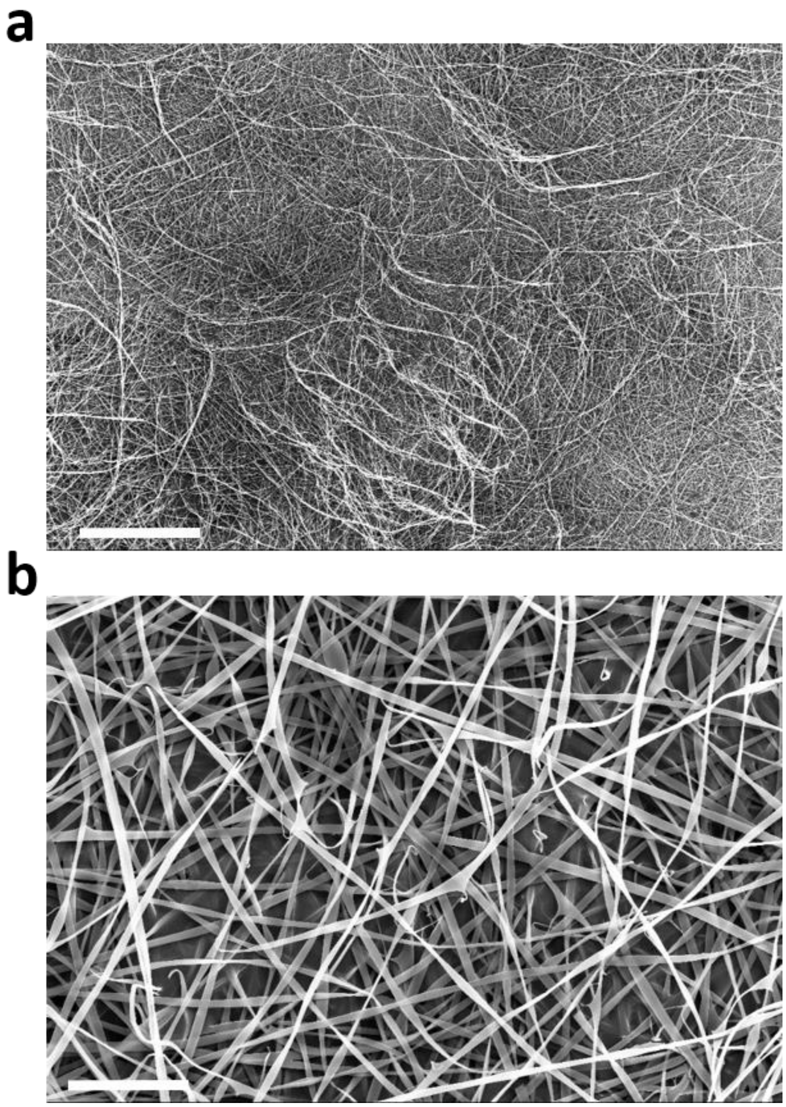

Supplementary Figure 14. SEM observation of fibroin interlays. a Low magnification and **b** high magnification SEM images of an electrospun fibroin interlayer. The SEM images of the interconnected structure of fibroin interlayers show rich pore structure, contributing to its excellent flexibility and excellent wettability towards electrolyte. Scale bars, 10 μm.

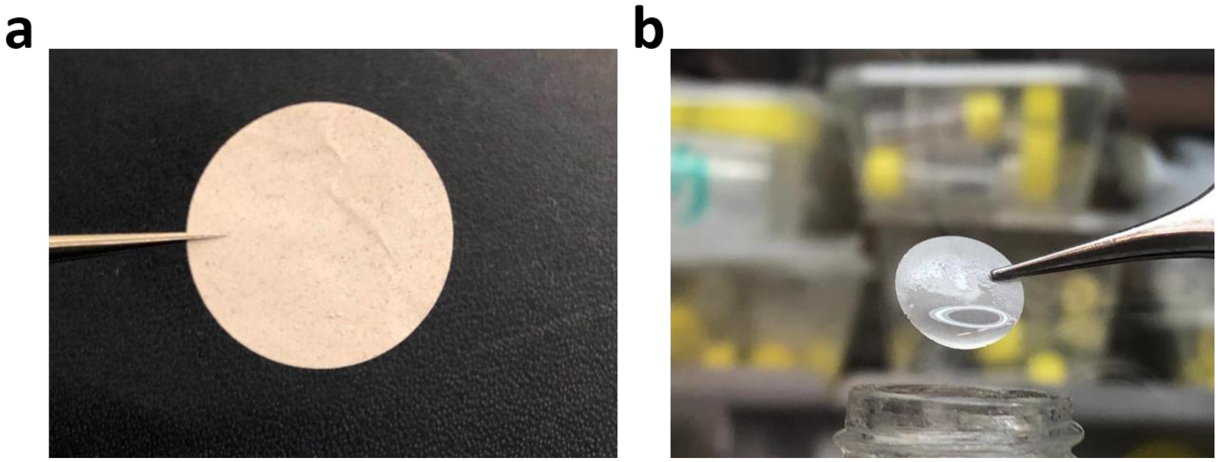

Supplementary Figure 15. Stability test of fibroin interlayer in ether-based electrolyte. a, **b** Digital photos of (**a**) the pristine fibroin interlayer and (**b**) the fibroin interlayer retrieved from electrolyte after immersed for 3 days.

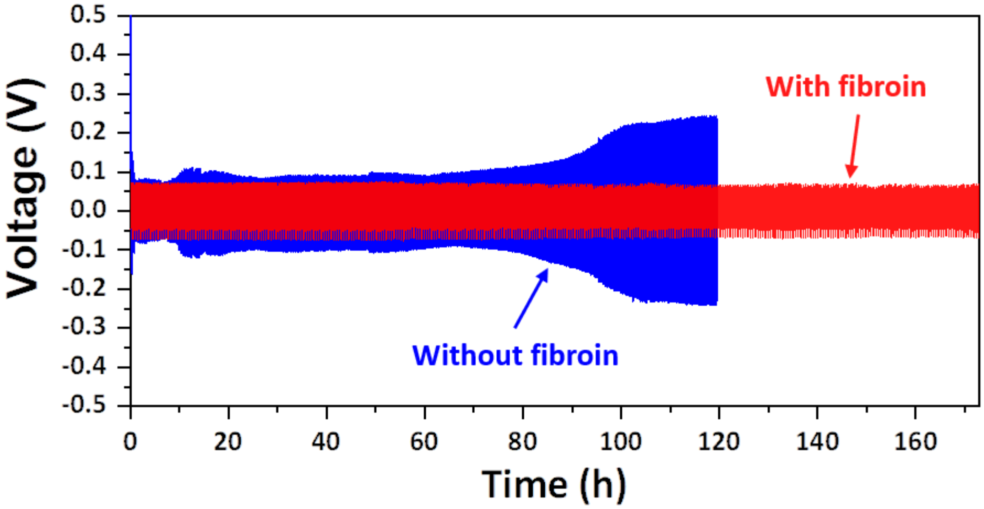

Supplementary Figure 16. The cycling stability of Li | Li symmetrical cells at a high current density. A current density of 5 mA cm^{-2} and a capacity of 1 mAh cm^{-2} were applied.

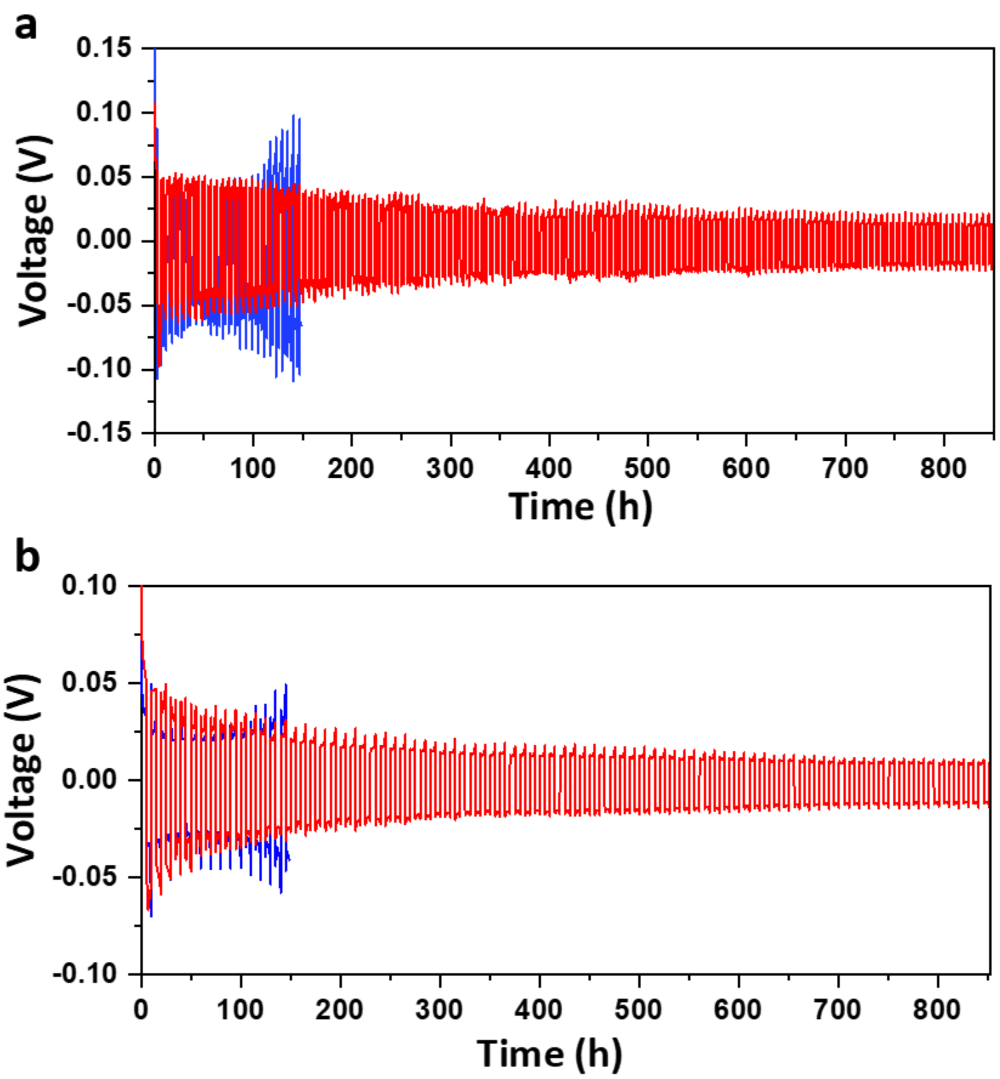

Supplementary Figure 17. Comparison of the cycling stability of Li | Li symmetrical cells at high capacities. a The capacity limitation is 3 mAh cm^{-2} and the current density is 1 mA cm^{-2}. **b** The capacity limitation is 5 mAh cm^{-2} and the current density is 1 mA cm^{-2}.

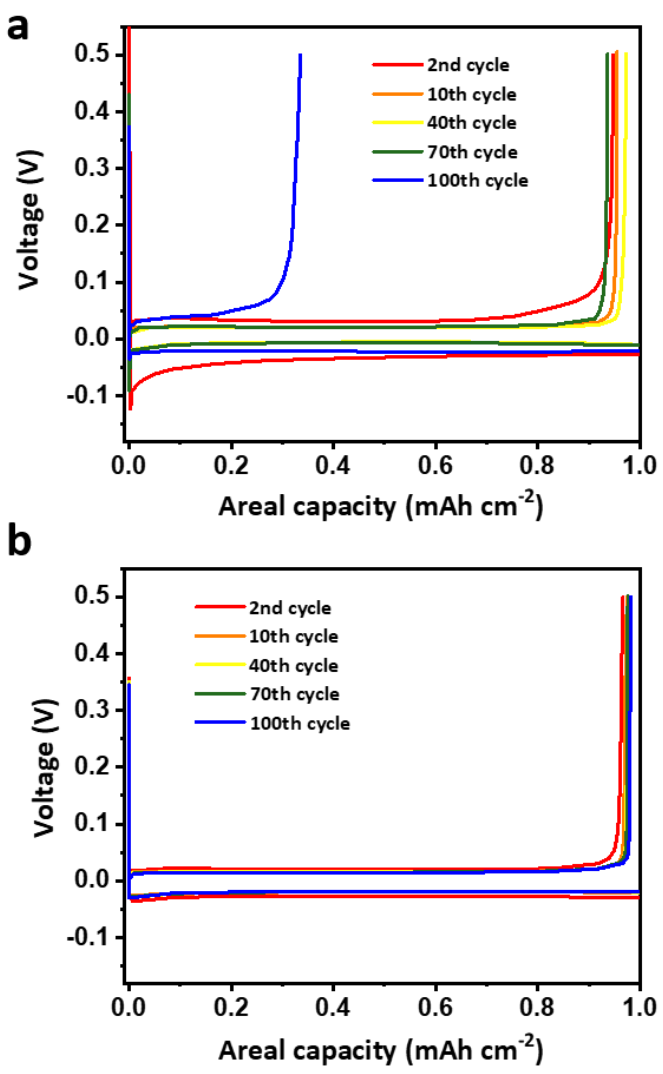

Supplementary Figure 18. Comparison of the cycling performances of Li | Cu half cells. a, **b** The voltage profiles of Li plating/striping on Cu foils at different cycles (**a**) without and (**b**) with fibroin interlayer.

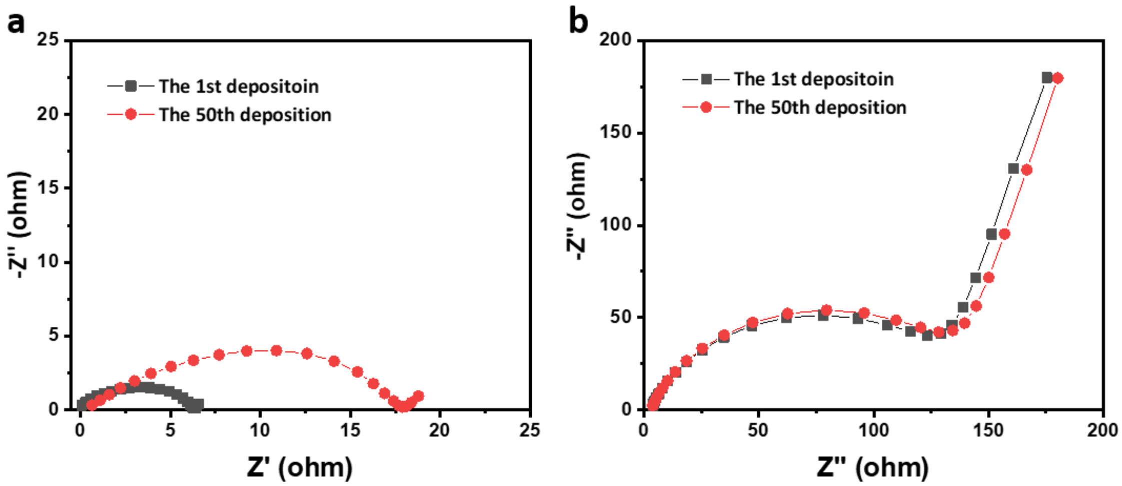

Supplementary Figure 19. The EIS measurement of Li | Cu half cells at different cycles. a without and **b** with the fibroin interlayer at the current density of 1 mA cm^{-2} with the capacity imitation of 1 mAh cm^{-2}.

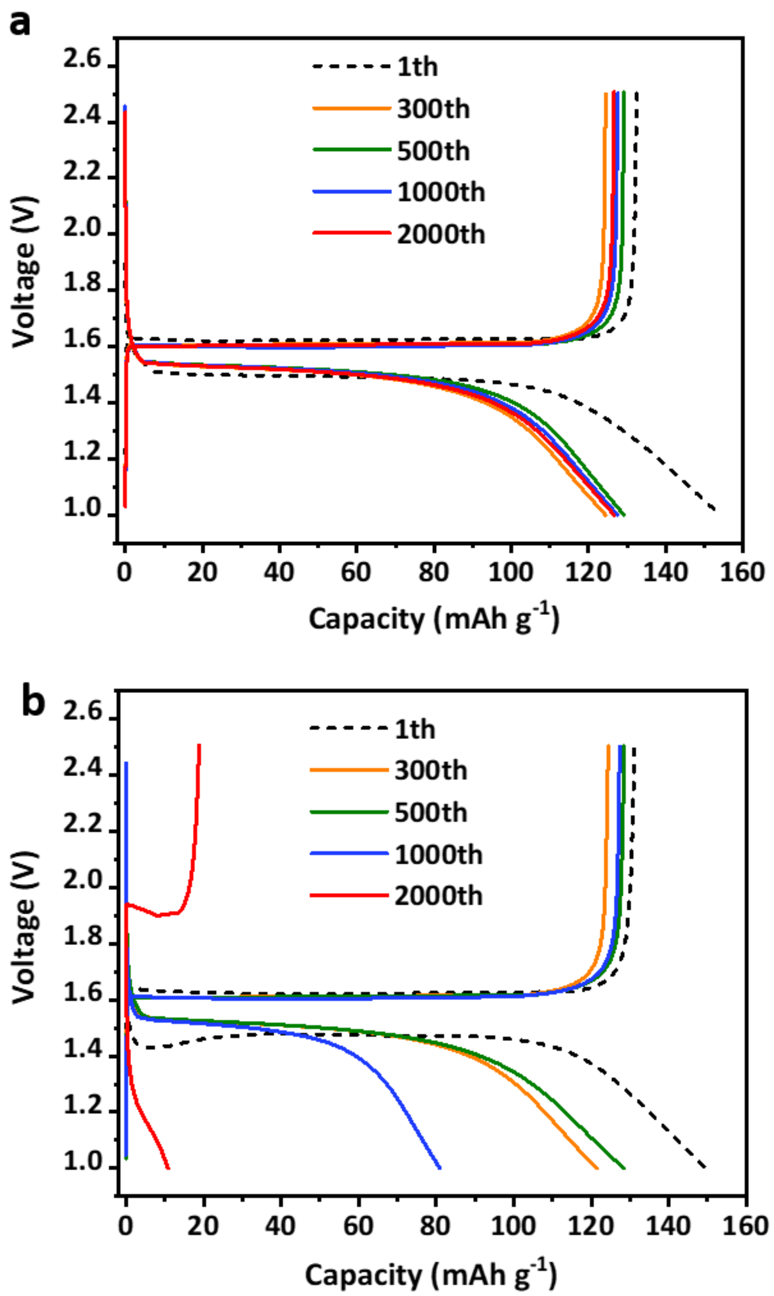

Supplementary Figure 20. Cycling performance of Li || LTO full cells. a, **b** Galvanostatic charge/discharge curves of Li || LTO full cells (**a**) with and (**b**) without fibroin interlayer.

Supplementary Table 1. Comparison of mechanical properties of fibroin interlayer before and after immersed in ether-based electrolyte.

Fibroin interlayer	Tensile strength (Mpa)	Elongation at break (%)	Young's modulus (Mpa)
Dry electrolyte	8.1 ±2.4	18.1 ±2.5	167 ±35
After immersed in electrolyte for 3 days	12.9 ±3.1	24.2 ±1.7	115 ±12

Supplementary Methods

Cryogenic TEM (Cryo-EM) characterizations. Cryo-EM characterizations were carried out using an FRI Titan 80-300 environmental scanning transmission electron microscope with an accelerating voltage of 300 kV. The instrument was equipped with an aberration corrector in the objective lens, which was tuned before each sample analysis. To prepare the cryo-EM sample, coin cells were assembled with the working electrode attached with 300 mesh Cu transmission electron microscopy (TEM) grids. After Li metal was deposited onto the TEM grid, coin cells were immediately disassembled in an Ar-filled glovebox. The TEM grids were washed with DOL to remove Li salts. After drying, the TEM grid with plated Li was placed in an Eppendorf tube and transferred out of the glovebox. A slight positive pressure inside the Ar-filled glovebox (and thus the Eppendorf tube) prevented air from leaking into the tube. The sealed Eppendorf tube was plunged quickly into a bath of liquid nitrogen (LN_2). Then the frozen Eppendorf tube was quickly crushed with a bolt cutter while still immersed in LN_2 to expose the Li metal to the cryogen. The TEM grid was carefully mounted onto a TEM cryo-holder (Gatan 626) using a cryo-transfer station under LN_2 immersion. During transferring TEM cryo-holder into the TEM column (~1 second), a built-in shutter on the holder was closed to prevent Li contacting with air. A LN_2 dewar bottle was attached to the holder to maintain the sample at cryogenic temperature. In this way, the Li metal can be safely transferred from the coin cell to the TEM chamber. Once inside the TEM chamber, the sample was kept at -178 °C.

COMSOL™ simulation. the stationary equation of continuity is used to handle the stationary electric currents in conductive media. In a stationary coordinate system, the point form of *Ohm's law* states:

$$\mathrm{J} = \sigma \mathrm{E} + \mathrm{J_e} \qquad \text{Supplementary Equation (1)}$$

Where σ is the electrical conductivity (S m^{-1}), and J_e is an externally generated current density (A m^{-2}). The static form of the equation of continuity then states:

$$\nabla \cdot \mathrm{J} = -\nabla \cdot (\sigma \nabla \mathrm{V} - \mathrm{J_e}) = 0 \qquad \text{Supplementary Equation (2)}$$

To handle the current sources, we generalize the equation to:

$$-\nabla \cdot (\sigma \nabla \mathrm{V} - \mathrm{J_e}) = \mathrm{Q_j} \qquad \text{Supplementary Equation (3)}$$

In planar two-dimension stimulation, the Electric Currents interface assumes that the model has a symmetry, where the electric potential varies only in the *x* and *y* directions and is constant in the *z* direction. This implies that the electric field, E, is tangential to the *xy*-plane. The Electric Currents interface then follows the following equation, where *d* is the thickness in the *z* direction:

$$-\nabla \cdot d(\sigma \nabla \mathrm{V} - \mathrm{J_e}) = d\mathrm{Q_j} \qquad \text{Supplementary Equation (4)}$$
